# Composition and aggregation of extracellular polymeric substances (EPS) in hyperhaline and municipal wastewater treatment plants

**DOI:** 10.1038/srep26721

**Published:** 2016-05-25

**Authors:** Jie Zeng, Jun-Min Gao, You-Peng Chen, Peng Yan, Yang Dong, Yu Shen, Jin-Song Guo, Ni Zeng, Peng Zhang

**Affiliations:** 1 Key Laboratory of the Three Gorges Reservoir Region’s Eco-Environments of MOE, Chongqing University, Chongqing 400045, China; 2 Key Laboratory of Reservoir Aquatic Environment of CAS, Chongqing Institute of Green and Intelligent Technology, Chinese Academy of Sciences, Chongqing 400714, China

## Abstract

As important constituents of activated sludge flocs, extracellular polymeric substances (EPS) play significant roles in pollutants adsorption, the formation and maintenance of microbial aggregates, and the protection of microbes from external environmental stresses. In this work, EPS in activated sludge from a municipal wastewater treatment plant (M-WWTP) with anaerobic/anoxic/oxic (A^2^/O) process and a hyperhaline wastewater treatment plant (H-WWTP) with anaerobic/oxic (A/O) process were extracted by ultrasound method. The proteins and polysaccharides contents in EPS were determined by using a modified Lowry method and anthrone colorimetry respectively to analyze the detail differences in two types of WWTPs. Fourier transform-infrared spectroscopy and three-dimensional excitation-emission matrix fluorescence spectroscopy demonstrated proteins and polysaccharides were the dominant components of the two types of EPS, and the aromatic protein-like substances accounted for a larger proportion in EPS proteins. The results of the aggregation test indicated that EPS were good for the sludge aggregation, and the EPS in oxic sludge were more beneficial to sludge aggregation than that in anoxic sludge. Anoxic sludge EPS in H-WWTP showed a negligible effect on sludge aggregation. Comparative study on EPS of different tanks in the M-WWTP and H-WWTP was valuable for understanding the characteristics of EPS isolated from two typical wastewater treatment processes.

Microbial extracellular polymeric substances (EPS) are a complex high-molecular-weight mixture of polymers excreted by microorganisms, produced from cell lysis and adsorbed organic matter from wastewater^1^. The main components (e.g., polysaccharides, proteins, humic substances and nucleic acids) in EPS exhibit crucial effect on microbial adhesion and aggregation processes, and promote the formation and stability of microbial community structure^2^. EPS can reduce adverse effects of the toxic substances on the cell and can be as the carbon source and energy for the microbes in the absence of nutrients^3^,^4^. However, the production and composition of EPS is influenced by many typical factors, such as substrate type (carbon sources and carbon nitrogen ratio), operation condition (dissolved oxygen, shear forces, sludge retention time and hydraulic retention time), growth stage, and solution chemistry (pH, ionic strength and divalent cation) and toxic substance (drugs and heavy metals)^5^,^6^,^7^,^8^,^9^,^10^,^11^. Especially, high salinity is considered as a limiting factor in the application of biological wastewater treatment processes, whereas EPS are a key factor that some strains used to bind Na^+^ allowing their survival in high NaCl concentrations^12^. Therefore, it is essential to understand the roles of EPS through characterizing EPS in hyperhaline wastewater treatment plant. In this work,comparison of the EPS composition and aggregation properties in hyperhaline and municipal wastewater treatment plant was investigated to demonstrate the effect of salinity and dissolved oxygen on EPS characteristics.

In recent years, the investigations on high salinity wastewater treatment and the influence of dissolved oxygen on wastewater treatment effect have attracted many researchers. Salinity stress has a negative effect on the biological treatment process^9^,^10^. The high Na^+^ concentration in the wastewater could exert the salinity pressure on the microorganism so that inhibited many kinds of enzymes activity, and decreased the microbial cells activity^13^. The extracellular polysaccharide generated amount reduced to a half when the Na^+^ 10 g/L increased to 20 g/L^14^. However, the amount of total EPS increased with the NaCl concentration changed from 0 to 10.0 g/L in a biofilm reactor, in which EPS mixture (e.g., the extracellular polysaccharide) secretion was considered to be a protective response to the bacteria in the salinity pressure^15^, as well as release of soluble microbial products and EPS also dramatically enhanced of the biomass to salinity stress in membrane bioreactor (MBR)^16^. The total EPS content of salt tolerant bacterium, *Rhodopseudomonas acidophila*, increased with the NaCl concentration, also suggesting a protective response of the bacterium to the salinity^11^. In terms of dissolved oxygen influence, Shin *et al.*^17^ found higher airflow rates increased the amount of carbohydrate in the bound EPS but the protein level was almost constant. Bayer and Ahimou *et al.*^18^,^19^ showed that high levels of oxygen promoting the production of polysaccharides. More bound EPS provided more opportunities for cells to become/remain embedded in EPS, so that free cells or small aggregates were less^20^. A higher microbial production of extracellular polymeric substances at a higher (4 mg/L) DO in high-loaded membrane bioreactors (HL-MBR) that gave a bigger mean floc size, a lower supernatant turbidity, better settleability and better membrane filterability than the HL-MBR that was operated at a DO of 1 mg/L^21^.

Although some researchers reported the effects of salinity or DO on EPS in pure cultures, activated sludges and biofilms, no systematic research has been carried out to distinguish EPS in a municipal wastewater treatment plant (M-WWTP) and a hyperhaline wastewater treatment plant (H-WWTP) with anaerobic/anoxic/oxic (A^2^/O) process and anaerobic/oxic (A/O) process respectively, as well as the aggregation sedimentation performance of activated sludge flocs in different tanks of these processes.

In order to reveal effects of dissolved oxygen and salinity on the composition and surface properties of EPS, in this work, EPS from the activated sludge in M-WWTP and H-WWTP were extracted by ultrasound method. The major functional groups of EPS were characterized using Fourier transform-infrared spectroscopy (FT-IR) and three-dimensional excitation-emission matrix (3D-EEM) fluorescence spectroscopy. In addition, the aggregation sedimentation performance of sludge flocs was investigated before and after EPS extraction as well.

## Results

### EPS main compositions’ contents analysis

According to previous research, the main components of the extracellular polymers were proteins and polysaccharides. In this work, the main components’ contents in EPS extracted from M-WWTP and H-WWTP was detected and the results were displayed in Table 1, which showed that the content of proteins in EPS extracted from M-WWTP with a descending order were anaerobic tank > secondary sedimentation tank > anoxic tank > oxic tank, while the polysaccharides content were anaerobic tank > anoxic tank > secondary sedimentation tank > oxic tank, and the value of PN/PS with a descending order were secondary sedimentation tank > oxic tank > anaerobic tank > anoxic tank. In H-WWTP system, the descending order of proteins contents in EPS were oxic tank > anaerobic tank > secondary sedimentation tank, while the polysaccharides content were anaerobic tank > secondary sedimentation tank > oxic tank, and the value of PN/PS with a descending order were oxic tank > anaerobic tank > secondary sedimentation tank. The total content of proteins and polysaccharides in EPS from H-WWTP was significantly higher than that of M-WWTP.

### FT-IR spectra characterization

**Figure 1:**
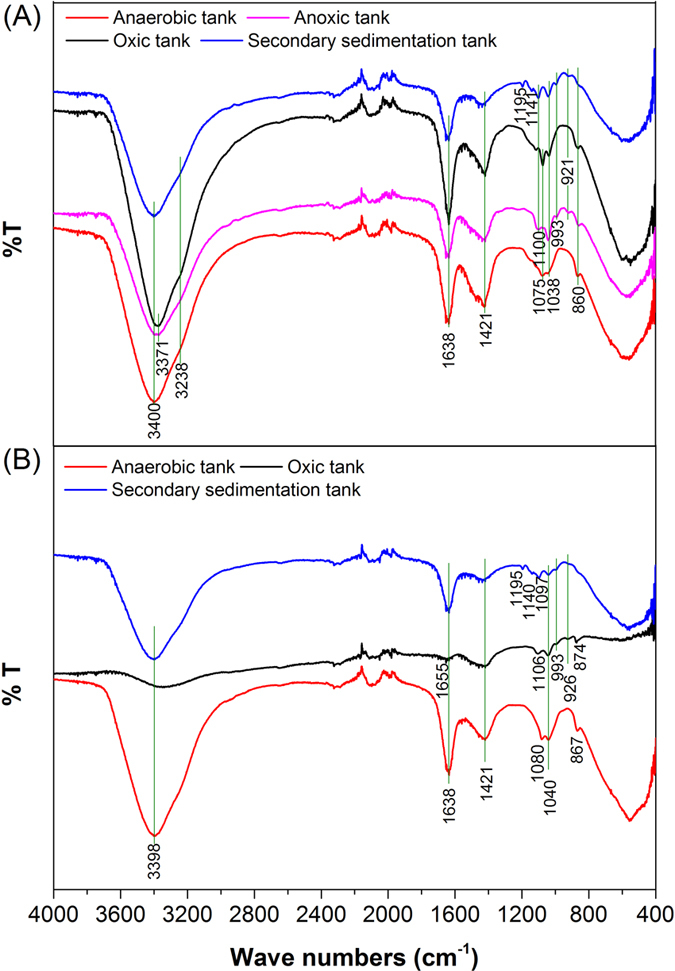
IR bands of the EPS from M-WWTP (**A**) and H-WWTP (**B**).

Figure 1 shows the infrared spectra of EPS from different sources. The infrared peaks were assigned according to the existing literatures. The broad peak at near 3400 cm^−1^ was specified for O–H stretching vibrations of polysaccharide^22^, whereas two peaks at 3371 cm^−1^ and 3238 cm^−1^ were related to N–H stretching vibrations of protein^22^ and C–H stretching vibrations^23^ . Peaks at 1655 cm^−1 ^and 1638 cm^−1^ were attributed to C=O stretching vibrations of protein amide groups with β-sheets structure and α-helix structure respectively^24^ , whereas peak at 1421 cm^−1^ was characteristic of C–H bending vibrations^22^. The peak between 921 and 1195 cm^−1^ were assigned to stretching vibrations of C–O and C–C, and deformation vibrations of C–O–H and C–O–C ,which also might be related to the vibrations of C–O–P , P–O–P or P=O^25^. It was the fingerprint region from 400 to 900 cm^−1^ and the peaks in the area were specified for phosphate groups or sulfated groups^26^.

Infrared spectra peaks of EPS from the oxic tank, anoxic tank and the secondary sedimentation tank for the M-WWTP activated sludge samples were very similar (Fig. 1A). The result showed that two peaks at 3371 cm^−1^ and 1638 cm^−1^ represented amino and α-helix of protein, respectively. It indicated that all the EPS in M-WWTP contained protein of α-helix structure. The bands of all EPS samples in M-WWTP between 1195 and 921 cm^−1^ had different absorption peaks, which implied that polysaccharide exited in each EPS from M-WWTP with various components.

The qualitative infrared spectra of EPS from the anaerobic, oxic and the secondary sedimentation tanks for the H-WWTP activated sludge samples exhibited difference (Fig. 1B). Figure 1B showed only three same EPS infrared peaks of anaerobic tank, oxic tank and the secondary sedimentation tank in H-WWTP. The peak of EPS from oxic tank in 1655 cm^−1^ and the peak of EPS from anaerobic tank and secondary sedimentation tank in 1638 cm^−1^ demonstrated each sample contains proteins with different secondary structure, and thus proteins in EPS of oxic tank were β-sheets structure while there were α-helix structure in anoxic tank and anoxic tank^24^. The infrared spectra ranging from 1195 to 926 cm^−1^ showed different absorption peaks in the three samples. It concluded that all the EPS in the anaerobic tank, oxic tank and the secondary sedimentation tank of H-WWTP contained polysaccharides, but their compositions were different.

### 3D-EEM fluorescence spectra characterization

**Figure 2:**
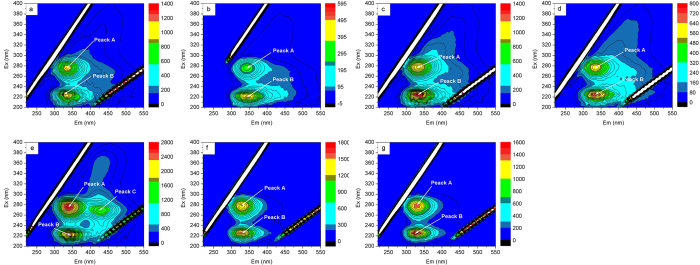
3D-EEM fluorescence spectra of the EPS from M-WWTP and H-WWTP (**a**) anaerobic tank in M-WWTP; (**b**) anoxic tank in M-WWTP; (**c**) oxic tank in M-WWTP; (**d**) secondary sedimentation tank in M-WWTP; (**e**) anaerobic tank in H-WWTP; (**f**) oxic tank in H-WWTP; (**g**) secondary sedimentation tank in H-WWTP).

Figure 2 showed the 3D-EEM spectra of EPS from M-WWTP and H-WWTP. There were two obvious fluorescence peaks A and B in the each EPS sludge sample. In addition to the two peaks, there was another fluorescence peak C in the EPS sample from anaerobic tank of H-WWTP. The fluorescence parameters of the spectra, including the peak locations and fluorescence intensities were summarized in Table 2. Peak A was observed at the excitation/emission wavelengths (Ex/Em) of 275–280/330–345 nm (tryptophan protein-like), and the peak B was identified at the Ex/Em of 220–225/325–355 nm (aromatic protein-like substances), while Peak C with low fluorescence intensity was identified at the Ex/Em of 270/425 nm (humic acid-like substances)^15^.

**Figure 3:**
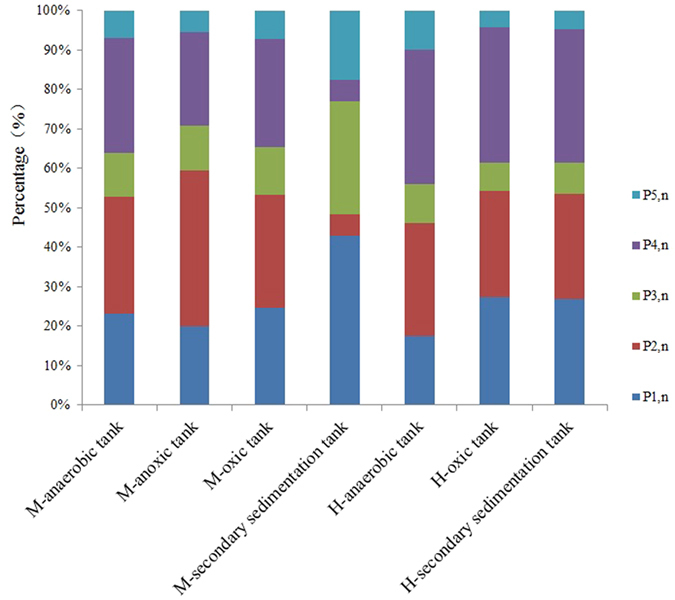
FRI percentage (Pi,n) values for 3D-EEM analysis of EPS samples from different tanks (M, M-WWTP; H, H-WWTP).

The FRI analysis results were showed in Table 3. The FRI percentage (Pi, n) values for 3D-EEM analysis of EPS samples from different tanks were displayed as Fig. 3. According to the results in Table 3, Fig. 3 and existing literatures^27^, it was concluded that aromatic protein-like substances (Regions I and II; P1, n and P2, n) were the main fluorescence matters existing in EPS of both M-WWTP and H-WWTP. Moreover, tryptophan protein-like substances (Region IV; P4, n) were the major fluorescence substances in EPS from each tank while the major substances in EPS from the secondary sedimentation tank in M-WWTP were fulvic acid-like materials (Region III, P3, n), and the percentages of tryptophan protein-like substances in EPS from H-WWTP were higher than from M-WWTP. In general, fulvic acid-like materials and humic acid-like organics (Region V; P5, n) were less than protein-like substances in EPS from every tank.

### Contribution of EPS to microbial cell aggregation

**Figure 4:**
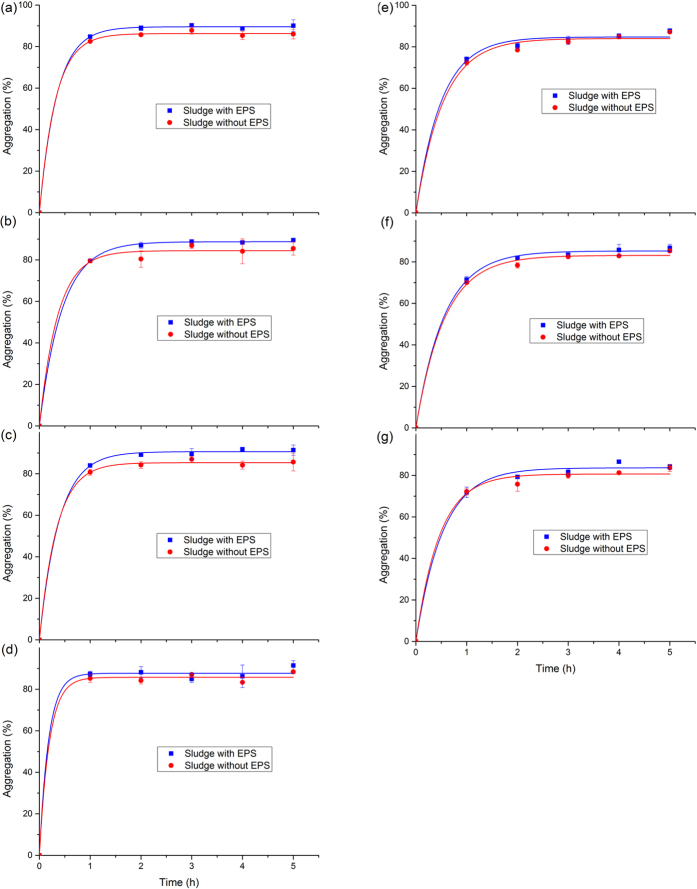
Aggregation ability of microbial cells suspension before and after EPS extraction from M-WWTP and H-WWTP (**a**) anaerobic tank in M-WWTP; (**b**) anoxic tank in M-WWTP; (**c**) oxic tank in M-WWTP; (**d**) secondary sedimentation tank in M-WWTP; (**e**) anaerobic tank in H-WWTP; (**f**) oxic tank in H-WWTP; (**g**) secondary sedimentation tank in H-WWTP).

Many investigations had indicated that EPS has an important influence on the adhesion and aggregation of microbial cells. Especially, the TB-EPS had the greatest contribution on the aggregation and settlement^28^, and the aggregation process was described by pseudo first-order dynamic equation^29^. In saline surroundings, microbes secreted more EPS and aggregated tightly to protect themselves both in MBR and A/O processes, and the sludge was quite compact^30^. Figure 4 showed the aggregation capability of microbial cells suspension before and after EPS extraction from M-WWTP and H-WWTP (a. anaerobic tank in M-WWTP; b. anoxic tank in M-WWTP; c. oxic tank in M-WWTP; d. secondary sedimentation tank in M-WWTP; e. anaerobic tank in H-WWTP; f. oxic tank in H-WWTP; g. secondary sedimentation tank in H-WWTP). It showed that the aggregation rates of each sample within 1 h were very high, whereas it almost could arrive at equilibrium state after 2 h. Besides, the aggregation rates of microbial cells suspension from the secondary sedimentation tank in M-WWTP, before and after EPS extraction, were much greater than that of other samples, which reached the equilibrium state less than 1 h. At the equilibrium state, the aggregation efficiency of each corresponding sample before EPS extraction was higher than after extraction, indicating that EPS was beneficial to the aggregation of microbial cells. It was consistent with the previous research^28^.

The kinetic parameters of microbial cell aggregations before and after extraction of EPS were listed in Table 4. The aggregation abilities of microbial cells suspension from M-WWTP (87.7~90.7%) were higher than from H-WWTP (83.7~85.3%) before EPS extraction. The *A*_e_ value decreased after EPS extraction, indicating the reduction of aggregation ability of microbial cells. The descending order of contribution of EPS in M-WWTP to aggregation were oxic tank (5.3%) > anoxic tank (4.2%) > anaerobic tank (3.3%) > secondary sedimentation tank (1.9%), whereas that of contribution of EPS in H-WWTP were secondary sedimentation tank (3%) > oxic tank (2.2%) > anaerobic tank (0.8%). In general, contribution of EPS on microbe aggregation ability in M-WWTP was more than that in H-WWTP. Especially, the impact of EPS on the aggregation of sludge in oxic tank, not only at M-WWTP but also at H-WWTP, was greater than in anaerobic tank. In addition, the EPS in anaerobic tank at H-WWTP had little influence on the aggregation and sedimentation of sludge. The reduction of microbial EPS aggregation could be related to the high salinity and anaerobic environment.

## Discussion

According to the results in EPS main compositions’ contents analysis, it could be concluded that proteins and polysaccharides accounted for significant proportion in EPS, and the contents of proteins and polysaccharides in EPS were different in any processing tank from the identical WWTPs. The reason resulted in the phenomena might be that the microbe external environment led to the diversity of microbial metabolism and species so that extracellular proteins and extracellular polysaccharides in EPS were distinct. In addition, the total content of proteins and polysaccharides in EPS from H-WWTP was significantly higher than that of M-WWTP when compared with the same type of processing tanks. It indicated that high salinity may promote cells to secrete much more EPS, which had a protective effect on the microbial cells as resistance to adverse environmental conditions to protect themselves and maintain the stability of the polymers.

There was some difference in infrared spectra of EPS between the two wastewater treatment systems. Compared with the infrared spectra (Fig. 1A,B), it exhibited that the infrared absorption peaks of EPS from M-WWTP were more abundant than those from H-WWTP, and the peak positions were also different, implying that the species of proteins and polysaccharides of EPS were distinct. It indicated that EPS components of M-WWTP were more complex. EPS strongly depend on microbial community and activity which are associated with two fundamental parameters, the ratios of food to microorganism (F/M) or dissolved oxygen (DO) levels^31^. In addition, high salt concentrations can induce salt stress to microbial species, resulting in the inhibition of various enzymes, decrease in cell activity, and plasmolysis^32^, which would cause the salt-tolerant bacteria as dominant species in H-WWTP and a certain degree of composition and amount difference of EPS between H-WWTP and M-WWTP secreted by different microbes finally.

According to the 3D-EEM spectra and FRI analysis results, it determined that protein-like substances were the main fluorescence substances existing in EPS of both M-WWTP and H-WWTP while a relatively small amount of humic acid-like and fulvic acid-like materials existed in all EPS samples even if kinds of fluorescence matters’ contents in EPS from each tanks were different. The high salinity could promote microbes secrete more fulvic acid-like materials so that contents of fulvic acid-like materials in EPS from M-WWTP were higher than from H-WWTP. The conclusions on compositions and contents in EPS from two typical WWTPs were of important significance on the latter study on EPS.

Based on results of aggregation assays, Liu *et al.*^33^ also found that oxic sludge had a higher aggregation rate than anaerobic sludge. High Na^+^ concentration could weaken the granularity of sludge flocs under high salinity, whereas only fragile and dispersive sludge produced^14^. Liao *et al.*^34^ illustrated that an increase in the DO level resulted in an increase in the total bound EPS, and there was an increasing trend in protein and total soluble EPS content with the thermophilic high DO condition. It was also reported that more EPS were generated in the aerobic region than in the anoxic or anaerobic regions^35^.

## Conclusions

The proteins and polysaccharides accounted for significant proportion in EPS, and the contents of proteins and polysaccharides in EPS were obviously different in any processing tank from the identical WWTPs. The total content of proteins and polysaccharides in EPS from H-WWTP was significantly higher than that of M-WWTP even though in the same type of processing tanks that indicated that high salinity may promote cells to secrete much more EPS. The FT-IR results also confirmed that all the EPS from both M-WWTP and H-WWTP comprised proteins and polysaccharides with different components. The EPS components of M-WWTP were more abundant. 3D-EEM spectra and FRI analysis results determined that protein-like substances were the main fluorescence substances existing in EPS of both M-WWTP and H-WWTP while a relatively small amount of humic acid-like and fulvic acid-like materials existed in all EPS samples and the high salinity could promote microbes secrete more fulvic acid-like materials. This work indicated that the impact of EPS on the aggregation of sludge in oxic tank, not only at M-WWTP but also at H-WWTP, was greater than in anaerobic tank. Furthermore, the EPS in anaerobic tank of H-WWTP had little influence on the aggregation and sedimentation of sludge. This investigation could be useful for controlling the adhesion and aggregation of microbial cells in M-WWTPs and H-WWTPs.

## Methods

### Activated sludge

Activated sludge samples were collected from the anoxic tank, anoxic tank, oxic tank and secondary sedimentation tank in Jiguanshi WWTP in Nan’an District of Chongqing, which is the fourth largest WWTP in China and the largest WWTP in Southwest China, serves a population of 1540000 equivalent inhabitants and treats up to 600000 m^3^/d of municipal wastewater^36^. High salinity activated sludges were collected from anaerobic tank, oxic tank, and secondary sedimentation tank of WWTP in the Yuquan mustard pickling industry in Wanzhou District, Chongqing, which adopts A/O process. The average salinity of influent is 9.8 g/kg and the average value of COD is 1500 mg/L. The concentration of ammonia nitrogen is 120 mg/L, while the average total nitrogen is 208 mg/L. The removal rate of COD, ammonia nitrogen and total nitrogen are about 92%, 95% and 66%, respectively.

### EPS extraction and chemical analysis

The EPS from activated sludges were extracted by ultrasonic method^37^. In general, 0.9% NaCl solution was used to clean the sludge for 3 times to remove the impurities in the sludge. The sludge mixed with 300 mL of 0.9% NaCl solution in a conical flask, and then treated by ultrasound at 20 kHz and 120 W for 3 min. The suspension was followed by constant temperature oscillation at 20 rpm for 10 min at 4 °C, and then the ultrasound was repeated again. The suspension was centrifuged at 10000 g for 30 min at 4 °C, and then filtered through 0.45 μm filters. The required EPS solution stored at 4 °C before analysis. The polysaccharides content was determined by anthrone colorimetry^27^. The proteins content was determined using a modified Lowry method^38^. The suspension volatile suspended solids (VSS) determination was according to the method described by Chinese NEPA^39^.

### Fourier transform infrared spectra (FT-IR)

The extracted EPS solution was freeze-dried at −80 °C and 200 mT. Dried EPS powders were analyzed using an FT-IR spectrometer (Cary 630, Agilent, USA).

### Three-dimensional excitation-emission matrix (3D-EEM) fluorescence spectroscopy

3D-EEM fluorescence spectra can be used to discriminate the fluorescence compounds present in EPS. 3D-EEM spectra of all EPS samples were measured using a luminescence spectrophotometer (F-7000 FL, Hitachi, Japan). In this study, the 3D-EEM spectra were collected with subsequent scanning emission spectra from 220 to 550 nm at 5 nm increments by varying the excitation wavelength from 200 to 400 nm at 5 nm sampling intervals. The excitation and emission slits were maintained at 5 nm. The scanning speed was set at 60000 nm min^−1^ for all the measurements. The 3D-EEM data were processed using the software Origin 9.0. Based on the 3D-EEM fluorescence spectra and the five excitation–emission regions as described by Chen^40^, fluorescence regional integration (FRI) analysis model was used to make the semi quantitative study on the different types of fluorescent matters in EPS.

### Aggregation assays

The role of EPS in the biological aggregation process was observed by an aggregation assay on microbial cells before and after EPS extraction. The developed method described by Eboigbodin and Biggs^41^ was performed to research the contribution of EPS to microbial cells aggregation. The cell pellet was resuspended in 0.9% NaCl (pH 7.0, 25 °C) before and after EPS extraction. The optical density (OD_600, 0_) of cell suspensions was adjusted to approximately 0.6, and then 4 mL of suspensions was transferred into the 5 mL cuvette. As the time elapsed, the cells aggregated and settled to the bottom of the cuvette, and the OD_600, t_ at the upper part of the cuvette was determined after 1, 2, 3, 4, and 5 h, respectively. All assays were in performed duplicate. The percentage aggregation (*A*_t_) of microbial cells was calculated using Eq. (1).


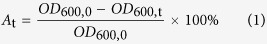


The aggregation process can be taken as a reversible chemical reaction. The kinetic of aggregation was described by pseudo first-order equation (Eq. (2)).


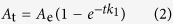


where *k*_1_ is the pseudo first-order rate constant (h^−1^), *A*_e_ is the percentage aggregation at equilibrium, and *A*_t_ is the percentage aggregation at a certain time (%)^28^.

## Additional Information

**How to cite this article**: Zeng, J. *et al.* Composition and aggregation of extracellular polymeric substances (EPS) in hyperhaline and municipal wastewater treatment plants. *Sci. Rep.* **6**, 26721; doi: 10.1038/srep26721 (2016).

## Figures and Tables

**Table 1:** Contents of the dominant components in EPS from M-WWTP (M) and H-WWTP (H).

Sludge	EPS		
	PN(mg/g VSS)	PS(mg/g VSS)	PN/PS
M-anaerobic tank	42.26	43.93	0.96
M-anoxic tank	36.86	40.51	0.91
M-oxic tank	34.94	35.52	0.98
M-secondary sedimentation tank	41.09	38.65	1.06
H-anaerobic tank	56.75	59.74	0.95
H-oxic tank	68.63	48.41	1.01
H-secondary sedimentation tank	46.77	54.38	0.86

**Table 2:** Fluorescence spectra parameters of the EPS from M-WWTP and H-WWTP.

Samples	Peak A		Peak B		Peak C		A/B
	Ex/Em	Intensity	Ex/Em	Intensity	Ex/Em	Intensity	
M-anaerobic tank	275/335	983.3	225/325	1192	–	–	0.82
M-anoxic tank	275/345	289.6	220/345	518.7	–	–	0.56
M-oxic tank	275/330	1122	225/355	1399	–	–	0.80
M-secondary sedimentation tank	275/330	600	225/335	744.3	–	–	0.81
H-anaerobic tank	275/340	2716	220/345	2605	270/425	1449	0.84
H-oxic tank	280/330	1523	225/330	1588	–	–	0.96
H-secondary sedimentation tank	280/330	1412	225/325	1587	–	–	0.89

**Table 3:** FRI volumetric (values (×10^7 ^AU nm^2^ (mg/L)^−1^) for 3D-EEM of EPS samples from M-WWTP and H-WWTP.

Samples	F_1,n_	F_2,n_	F_3,n_	F_4,n_	F_5,n_	Summation
M-anaerobic tank	1.33	1.70	0.64	1.68	0.40	5.75
M-anoxic tank	0.40	0.80	0.23	0.48	0.11	2.02
M-oxic tank	1.80	2.09	0.88	2.01	0.53	7.31
M-secondary sedimentation tank	8.71	1.15	5.83	1.11	3.57	20.37
H-anaerobic tank	2.27	3.75	1.32	4.47	1.29	13.10
H-oxic tank	1.79	1.75	0.48	2.24	0.28	6.54
H-secondary sedimentation tank	1.68	1.67	0.50	2.12	0.30	6.27

**Table 4:** Kinetic parameters of microbial cells aggregation before and after EPS extractions (M, M-WWTP; H, H-WWTP).

Sludge M-anaerobic tank	*k*_1_ (h^−1^)		*A*_e_ (%)			*R*^2^	
	With EPS	Without EPS	With EPS	Without EPS	*∆*	With EPS	Without EPS
M-anaerobic tank	2.9	3.1	89.6	86.3	3.3	0.999	0.999
M-anoxic tank	2.2	2.6	88.6	84.4	4.2	0.999	0.995
M-oxic tank	2.6	2.9	90.7	85.4	5.3	0.999	0.999
M-secondary sedimentation tank	5.5	5.1	87.7	85.8	1.9	0.995	0.997
H-anaerobic tank	2.0	1.9	84.8	84.0	0.8	0.996	0.994
H-oxic tank	1.8	1.8	85.3	83.1	2.2	0.999	0.995
H-secondary sedimentation tank	1.9	2.2	83.7	80.7	3.0	0.996	0.994
